# Magnetoliposomes Based on Magnetic/Plasmonic Nanoparticles Loaded with Tricyclic Lactones for Combined Cancer Therapy

**DOI:** 10.3390/pharmaceutics13111905

**Published:** 2021-11-10

**Authors:** Irina S. R. Rio, Ana Rita O. Rodrigues, Juliana M. Rodrigues, Maria-João R. P. Queiroz, R. C. Calhelha, Isabel C. F. R. Ferreira, Bernardo G. Almeida, Ana Pires, André M. Pereira, João P. Araújo, Elisabete M. S. Castanheira, Paulo J. G. Coutinho

**Affiliations:** 1 Centre of Physics of Minho and Porto Universities (CF-UM-UP), University of Minho, Campus de Gualtar, 4710-057 Braga, Portugal; id9261@alunos.uminho.pt (I.S.R.R.); ritarodrigues@fisica.uminho.pt (A.R.O.R.); bernardo@fisica.uminho.pt (B.G.A.); 2 Centre of Chemistry (CQUM), University of Minho, Campus de Gualtar, 4710-057 Braga, Portugal; juliana.mourarodrigues@gmail.com (J.M.R.); mjrpq@quimica.uminho.pt (M.-J.R.P.Q.); 3 Mountain Research Centre, Polytechnic Institute of Bragança, Campus de Santa Apolónia, 5300-253 Bragança, Portugal; calhelha@ipb.pt (R.C.C.); iferreira@ipb.pt (I.C.F.R.F.); 4 IFIMUP—Instituto de Física dos Materiais, Universidade do Porto, R. Campo Alegre, 4169-007 Porto, Portugal; ana.pires@fc.up.pt (A.P.); ampereira@fc.up.pt (A.M.P.); jearaujo@fc.up.pt (J.P.A.)

**Keywords:** manganese ferrite/gold nanoparticles, magnetoliposomes, new antitumor drugs, thienopyridine derivatives, combined cancer therapy

## Abstract

Liposome-like nanoarchitectures containing manganese ferrite nanoparticles covered or decorated with gold were developed for application in dual cancer therapy, combining chemotherapy and photothermia. The magnetic/plasmonic nanoparticles were characterized using XRD, UV/Visible absorption, HR-TEM, and SQUID, exhibiting superparamagnetic behavior at room temperature. The average size of the gold-decorated nanoparticles was 26.7 nm for MnFe_2_O_4_ with 5–7 nm gold nanospheres. The average size of the core/shell nanoparticles was 28.8 nm for the magnetic core and around 4 nm for the gold shell. Two new potential antitumor fluorescent drugs, tricyclic lactones derivatives of thienopyridine, were loaded in these nanosystems with very high encapsulation efficiencies (higher than 98%). Assays in human tumor cell lines demonstrate that the nanocarriers do not release the antitumor compounds in the absence of irradiation. Moreover, the nanosystems do not cause any effect on the growth of primary (non-tumor) cells (with or without irradiation). The drug-loaded systems containing the core/shell magnetic/plasmonic nanoparticles efficiently inhibit the growth of tumor cells when irradiated with red light, making them suitable for a triggered release promoted by irradiation.

## 1. Introduction

Cancer is a major public health problem, and the World Health Organization (WHO) points to it as the second leading cause of death worldwide. In 2018, nearly 9.6 million people died from several forms of cancer and in 2019, 1,762,450 new cancer cases were diagnosed and 606,880 deaths occurred in the United States. These numbers correspond to 4800 new cases and 1700 deaths per day [1]. One of the most prominent difficulties in cancer treatment is to achieve high concentrations of chemotherapeutic drugs in the malignant site while reducing nonspecific distribution throughout healthy tissues. In this regard, stimuli-responsive nanosystems for target therapy have been a promising approach to control the release and delivery of therapeutic agents at the target sites. In addition, it is equally important to improve the drug therapeutic effect on tumors, and combination therapies have been suggested [2,3]. The idea is to achieve an appropriate synergistic effect between two or more treatment methodologies, such as chemotherapy, hyperthermia, and/or radiotherapy, to decrease drug resistance, and improve the therapeutic effect of antitumor compounds by chemosensitization of tumor cells [4].

Multifunctional nanosystems are one of the most promising therapeutic approaches in cancer treatment [3,5]. The possibility of combining liposomes with magnetic nanoparticles stands out, allowing theranostic capabilities by simultaneous magnetic guidance to the tumor, combination therapy, and magnetic resonance imaging in a single nanosystem [6,7,8]. Regarding magnetic nanoparticles, transition metal ferrites, MFe_2_O_4_ (with M = Fe, Ni, Co, Mn), have been the most used. These ferrites are soft-magnetic materials with great superparamagnetic behavior, chemical stability, and mechanical hardness [9,10,11]. Manganese ferrite nanoparticles are particularly interesting due to the higher magnetic susceptibility compared to the other transition metal ferrites [11,12]. However, toxicity issues have been a concern, and recent investigations recommend the use of nanomaterials with improved biocompatibility [13]. Therefore, organic or inorganic shells have been used to reduce toxicity and control the bioaccumulation, bioelimination, and undesired tissue invasion [14], though it might hinder the preservation of magnetic properties of the core material [15]. The coverage of magnetic nanoparticles with a gold layer offers increased biocompatibility while allowing additional plasmonic features, providing a multifunctional material bearing magnetic and plasmonic capabilities. The easy surface functionalization, low toxicity, and the surface plasmon resonance (SPR) phenomenon have contributed to the use of gold nanoparticles in several biomedical applications [16,17,18]. In fact, gold-based nanostructures have found extensive use in different technological areas, such as immunologic assays, biosensors, photothermolysis of tumor cells, the detection and control of microbes and drug nanocarriers [19,20,21,22,23,24]. Gold nanoparticles can be used in photothermal therapy, as they can efficiently convert light into heat. In cancer treatment, this photothermal effect can be used to induce a local temperature increase and, consequently, hyperthermia physiological responses that lead to tumor cell death [22]. Recently, a synergistic cytotoxic effect has been observed by the combination of magnetic hyperthermia with phototherapy [25].

**Figure 1:**
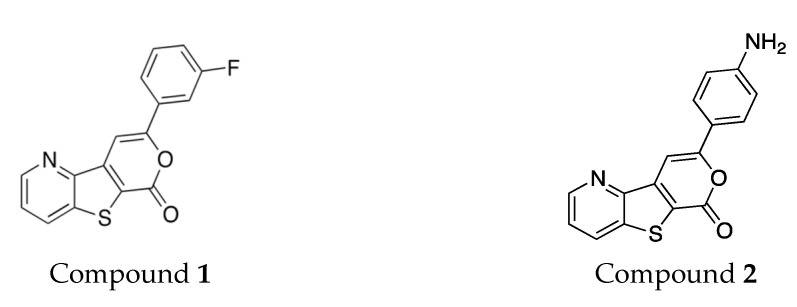
Structure of the tricyclic lactone derivatives of thienopyridine.

In this work, magnetic nanoparticles of manganese ferrite were decorated with gold nanoparticles or covered by a gold shell. The prepared magnetic/plasmonic nanostructures were entrapped in liposomes and the multifunctional nanosystems were tested as nanocarriers for two new potential antitumor thienopyridine derivatives. Thienopyridine compounds have been described as inhibitors for receptor tyrosine kinase and promising anticancer agents [26,27,28]. The new compounds, tricyclic lactones (Figure 1), have shown promising antitumor activity [28], exhibiting very low inhibitory concentrations in human colorectal adenocarcinoma (HCT-15 cell line) and non-small cell lung cancer (NCI-H460 cell line) (Table 1). These compounds were incorporated in the magnetic/plasmonic nanosystems and the antiproliferative activity was tested in vitro against the human tumor cell line NCI-H460.

## 2. Materials and Methods

All solutions were prepared using spectroscopic -grade solvents and ultrapure water of Milli-Q grade (MilliporeSigma, St. Louis, MO, USA).

### 2.1. Synthesis of Manganese Ferrite/Gold Nanoparticles

#### 2.1.1. Manganese Ferrite Nanoparticles Preparation

Manganese ferrite nanoparticles (NPs) were produced through the coprecipitation method. Briefly, to a 5 mL aqueous solution that contained 612 µL of 50% NaOH (product number 43617 from Sigma-Aldrich, St. Louis, MO, USA) at 90 °C, 1 mL of a mixture of 0.25 M MnSO_4_.H_2_O (product number 1.05941 from Sigma-Aldrich, St. Louis, MO, USA) and 0.5 M FeCl_3_.6H_2_O (product number 236489 from Sigma-Aldrich, St. Louis, MO, USA) was added, drop by drop, under magnetic stirring. After two hours at 90 °C, manganese ferrite nanoparticles (MnFe_2_O_4_) were obtained. For purification, the obtained nanoparticles were washed several times by repeated centrifugations and magnetic decantation, followed by drying at 100 °C.

#### 2.1.2. Gold Nanoparticles Preparation

A stable solution of gold nanoparticles in toluene was prepared following a method similar to that described by Brust et al. [29]. Briefly, an aqueous solution of gold (III) chloride hydrate (30 mM) (product number 254169, from Sigma-Aldrich, St. Louis, MO, USA) was mixed with tetraoctylammonium bromide (product number 426288 from Sigma-Aldrich, St. Louis, MO, USA) (50 mM) in toluene. The latter acts as a phase-transfer reagent, allowing the solubilization of gold ions in the toluene organic phase. Then, sodium borohydride (product number 632287 from Sigma-Aldrich, St. Louis, MO, USA) (0.4 M) was slowly added, under magnetic stirring. The obtained gold nanoparticles were washed with deionized water by centrifugation cycles.

#### 2.1.3. Synthesis of Gold-Seed Manganese Ferrite Nanoparticles

1,1′-carbonyldiimidazole (CDI) (product number 705284 from Sigma-Aldrich, St. Louis, MO, USA) was used to promote the covalent coupling of cysteamine to the hydroxyl surface groups of the manganese ferrite nanoparticles. Firstly, 5 mL of dried dioxane was used as the solvent for the coupling of 1 mg magnetic nanoparticles with a 5-fold molar excess of CDI over metals concentration, under sonication at 60 °C. After purification by centrifugation/sedimentation cycles using ethanol, cysteamine was added at the same 5-fold molar excess and the system was allowed to react for 1 h at 60 °C, under sonication. Upon washing with ethanol and drying in an oven, cysteamine-functionalized (product 8.02835 from Sigma-Aldrich, St. Louis, MO, USA) manganese ferrite nanoparticles were dispersed in toluene. Upon the addition of a diluted solution of gold nanoparticles in toluene (3 mL, 1:19.75) a color change occurred, which indicates the gold surface coupling with SH groups from cysteamine molecules pending on the surface of magnetic nanoparticles. This was further confirmed by solution color disappearance when the resulting nanoparticles were accumulated on the wall of the tube by the use of a small magnet. Finally, the manganese ferrite nanoparticles decorated with gold were washed with ethanol by magnetic decantation.

#### 2.1.4. Synthesis of Gold-Coated Manganese Ferrite Core-Shell Nanoparticles

Gold-coated manganese ferrite core-shell nanoparticles were prepared by the growth and coalescence of the gold-decorated nanoparticles as obtained in the previous section. The procedure was based on a previously reported “seeding” method [30]. Briefly, to a 1.5 mL aqueous solution of MnFe_2_O_4_ decorated with gold nanoparticles ([Au] = 0.6 mM), 2.4 mL of HAuCl_4_ (0.01%) and 100 µL of hydroxylamine (product number 255580 from Sigma-Aldrich, St. Louis, MO, USA) (40 mM) were added. Additional growth was promoted by the further addition of 100 µL of 1% HAuCl_4_. Purification was conducted by washing with water through magnetic decantation and, at the end, the particles were dispersed in ethanol. In order to monitor the gold shell formation, absorption spectra were measured in 10 s intervals.

### 2.2. Preparation of Magnetoliposome-Type Structures

For the preparation of magnetoliposome-type structures, the lipid DPPC (dipalmitoylphosphatidylcholine, product 850355P from Sigma-Aldrich, St. Louis, MO, USA) was used, in a final concentration of 2 mM. A solution of manganese ferrite/gold nanoparticles, resulting from the process in Section 2.1.3 or Section 2.1.4, in absolute ethanol (1.5 mL) was prepared. Then, octadecylamine (ODA) (product number 305391, from Sigma-Aldrich, St. Louis, MO, USA), in a 5-fold excess, was added to the nanoparticles, and this solution was stirred for one hour. The solution was washed, and successive centrifugation and magnetic decantation were employed, to remove unbound ODA. After this step, an ethanolic solution of DPPC was added to the solution of nanoparticles covered with ODA. After mixing and solvent evaporation, a lipid film was obtained. Then, ultrapure water was added, and the mixture was ultrasonicated, to promote self-assembling into magnetoliposome formation, followed by two washing steps to remove excess of lipids.

The antitumor tricyclic lactones **1** or **2** were incorporated into the liposome-type structures (containing the manganese ferrite/gold nanoparticles) by the injection of an ethanolic solution immediately before the formation of the lipid film step.

### 2.3. Preparation of Giant Unilamellar Vesicles (GUVs)

The thin-film hydration method was used to prepare the giant unilamellar vesicles of soybean lecithin (l-α-phosphatidylcholine from soybean) (product number 524617 from Sigma-Aldrich, St. Louis, MO, USA). The methodology described by Tamba et al. was employed, consisting of a pre-hydration of the soybean lecithin (1 mM) thin film with 40 µL of water and incubation at 45 °C for 30 min, followed by the addition of 3 mL of a glucose (product number G8270 from Sigma Aldrich, St. Louis, MO, USA) aqueous solution (0.1 M) and incubation at 37 °C for 2 h [31,32]. In order to remove multilamellar vesicles and lipid aggregates, the suspension was centrifuged for 30 min under 14,000× *g*.

### 2.4. Spectroscopic Measurements

#### 2.4.1. Absorption and Fluorescence Spectra

The absorption and emission spectra were obtained using the spectrophotometer Shimadzu UV-3600 Plus UV-vis-NIR (Shimadzu Corporation, Kyoto, Japan) and the spectrofluorimeter Horiba Fluorolog 3 (HORIBA Jobin Yvon IBH Ltd., Glasgow, UK), respectively. The latter included a temperature-controlled sample cuvette holder, double monochromators in excitation and emission, and Glan-Thompson polarizers (for fluorescence anisotropy measurements). 

The steady-state fluorescence anisotropy, *r*, is calculated by the equation below.

(1) $$r=\frac{I_{\mathrm{VV}}-GI_{\mathrm{VH}}}{I_{\mathrm{VV}}+2GI_{\mathrm{VH}}}$$

In Equation (1), *I*_VV_ and *I*_VH_ represent the fluorescence intensities obtained with vertical or horizontal polarization using vertically polarized excitation light, respectively. The instrument correction factor (G) is obtained by the ratio $I_{\mathrm{HV}}/I_{\mathrm{HH}}$, where *I*_HV_ and *I*_HH_ are the corresponding emission intensities using horizontally polarized excitation light.

#### 2.4.2. Drug Encapsulation Efficiency

Encapsulation studies of the potential antitumor drugs in the nanocarriers were performed using Amicon^®^ Ultra centrifugal filter units 100 kDa (Merck Millipore, Darmstadt, Germany) using 60 min centrifugation at 11,000 rpm. Upon solvent evaporation and redispersion in ethanol, the amount of non-encapsulated dye was determined through fluorescence emission using a calibration curve. The encapsulation efficiency (*EE*%) is then obtained by:

(2) $$EE\left(\%\right)=\frac{\left(total\;amount-amount\;of\;non\;encapsulated\;compound\right)}{total\;amount}\times 100$$

The *EE*(%) standard deviation (SD) was calculated using three independent measurements. 

### 2.5. Characterization of Structural and Magnetic Properties 

Electron microscopy images of the synthesized nanoparticles and nanosystems were obtained in a JEM 2010F high-resolution transmission electron microscope (HR-TEM) from JEOL Ltd. operating at 200 kV, at C.A.C.T.I (Centro de Apoio Científico e Tecnolóxico á Investigación), Vigo, Spain. STEM images were recorded using a NanoSEM–FEI Nova 200 (FEI Company, Hillsboro, OR, USA), operating at 15 kV, coupled to an Electron Dispersive Spectroscopic analyzer (EDS) and Electron Backscatter Diffraction EDAX—Pegasus X4M analyzer (AMETEK Inc., Berwyn, PA, USA) and detection system (EBSD) at SEMAT (Serviços de Caracterização de Materiais, Guimarães, Portugal). The samples were prepared by placing a drop of a solution-dispersed material onto a copper grid with Formvar/carbon (Agar Scientific Ltd., Essex, UK) and drying. The ImageJ software (National Institutes of Health (NIH), version 1.53c, Bethesda, MD, USA), was used for the manual selection of ellipsoidal contours from which particle diameters were calculated using the particles area. The corresponding histograms were fitted to Gaussian distributions. Dynamic light scattering (DLS) measurements at 25 °C and a 173° detector angle were obtained using NANO ZS Malvern Zetasizer (Malvern Panalytical Ltd., Malvern, UK) equipped with a He-Ne laser (λ = 632.8 nm). These measurements allowed the estimation of the magnetoliposomes-type nanosystem’s mean diameter and size distribution. For each sample, five independent measurements were performed. The crystallography measurements were performed in an X-ray diffraction (XRD) diffractometer equipment PAN’alytical X’Pert PRO diffractometer (Malvern Panalytical Ltd., Malvern, UK), operating in a Bragg-Brentano configuration using CuK_α_ radiation. Magnetic properties were investigated using a superconducting quantum interference device (SQUID) magnetometer Quantum Design MPMS5XL (Quantum Design Inc., San Diego, CA, USA), operating with an up to 5.5 T applied magnetic field.

### 2.6. Measurement of the Photothermal Effect

**Scheme 1:**
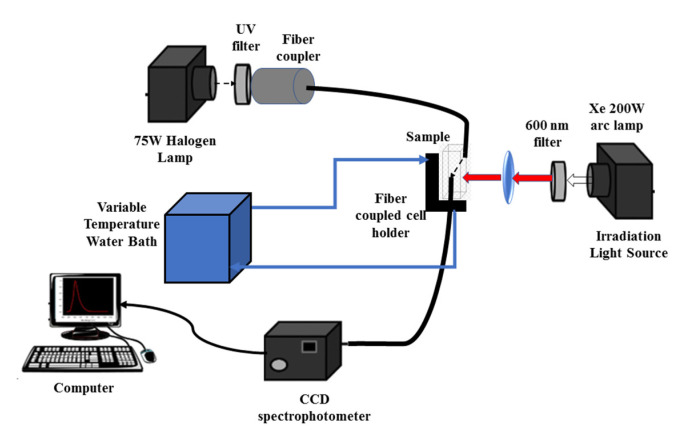
Representation of the photothermal effect measurement setup.

The irradiation source consisted of a Xenon arc lamp (200 W) focused through a Thorlabs FEL0600 (Thorlabs Inc., Newton, NJ, USA) long-pass filter with a cut-on wavelength at 600 nm, on a cell holder with fiber adaptors. In this way, only gold nanoparticles absorb the irradiation light, with minimal excitation of the dye Nile Red. Magnetoliposomes incorporating Nile Red were divided in two aliquots. For one of these aliquots, absorption spectra were measured at several temperatures by the use of a water bath. For the other, absorption spectra were measured after several irradiation times. The experimental setup is represented in Scheme 1. The monitoring channel used a 75 W halogen bulb and a compact CCD optical fiber spectrophotometer (350–700 nm, Δλ < 0.5 nm, slit = 20 µm, CCS100 Thorlabs Inc., Newton, NJ, USA) with 20 ms acquisition time and 100 averages. In order to avoid the impact of scattered light from the irradiation lamp, the irradiation optical channel was blocked during the acquisition of the monitoring light intensity. An aqueous dispersion with P25 TiO_2_ nanoparticles, with approximately the same scattering effect as the magnetoliposomes solution, was used as a reference. 

### 2.7. Studies in Cell Lines

The in vitro cytotoxicity of the magnetoliposomes loaded with compounds **1** and **2**, with different concentrations (2.28 µM to 146 µM, for compound **1** and 3.50 µM to 228 µM, for compound **2**), was evaluated in the primary non-tumor cell line PLP2 and a non-small cell lung cancer line (NCI-H460). Cells were obtained from the European Collection of Cell Cultures (Salisbury, UK). The in vitro effect on the growth of cell lines was evaluated according to the procedure adopted by the National Cancer Institute (NCI) (Bethesda, MD, USA) in the “*In vitro Anticancer Drug Discovery Screen*” using the protein-binding dye sulforhodamine B (SRB) to assess cell growth [33,34].

The cells were maintained in a humidified air incubator containing 5% CO_2_, at 37 °C, as adherent cell cultures in the RPMI-1640 medium containing 10% heat-inactivated FBS. Every cell line was plated to a density of 1.0 × 10^4^ cells per well in 96-well plates and allowed to attach for 24 h. After that, the cells were treated for 48 h with different solutions. After this incubation period, the adherent cells were fixed by adding cold 10% trichloroacetic acid (TCA) (100 μL) and incubated for 60 min at 4 °C. Finally, the plates were washed with deionized water and dried. Then, an SRB assay was used. A 100 mL SRB solution (0.1% in 1% acetic acid) was placed in each plate-well and incubated at room temperature for half an hour. The plates were washed with 1% acetic acid solution to remove unbound SRB. After drying, 200 µL of a 10 mM Tris-HCl buffer was added to the SRB bound in the plates and the absorbance was measured in a microplate reader at 540 nm to estimate the GI_50_ values (concentration that inhibited 50% of net cell growth).

**Figure 2:**
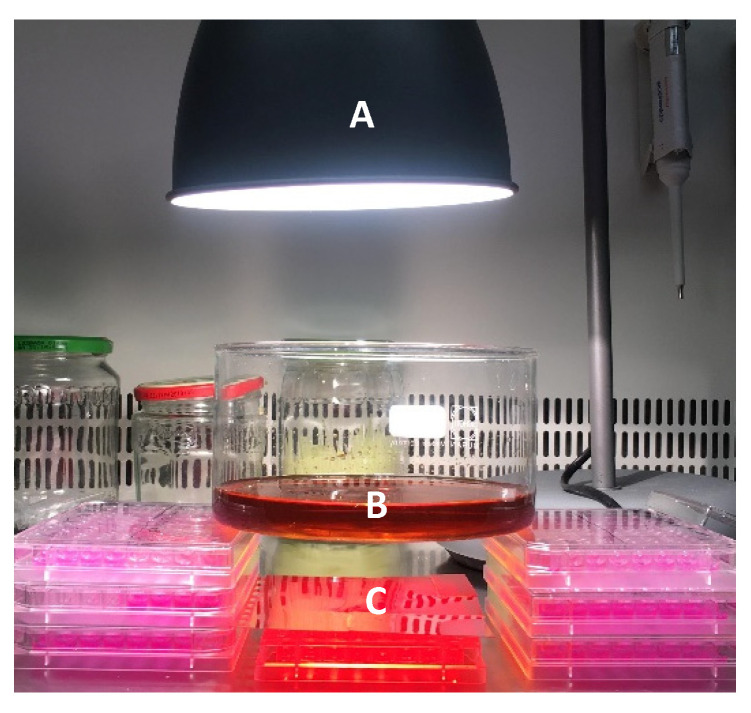
In vitro assays using cell lines under irradiation. (**A**) Halogen/tungsten lamp; (**B**) aqueous solution of iron(III) nitrate, (**C**) 96-well plates and cut-off filter to remove light of wavelength below ~500 nm.

For the cytotoxicity assays of the drug-loaded magnetoliposomes under irradiation, a halogen/tungsten lamp (24 V and 250 W, OSRAM, Porto, Portugal) with a fluence rate of 19–20 µW/cm^2^ (measured with an ILT 1400-A radiometer equipped with one SEL033 detector (ILT, Peabody, MA, USA) was used (Figure 2). Cells were continuously irradiated for 30 min followed by 24 h after incubation. A 1 M aqueous iron(III) nitrate solution was placed between the lamp and the cells as a liquid cut filter to remove the light of wavelengths less than ~500 nm [35]. The temperature the cells were exposed to was carefully monitored to ensure cell viability.

## 3. Results and Discussion

### 3.1. Photophysical Properties of the Antitumor Compounds

**Figure 3:**
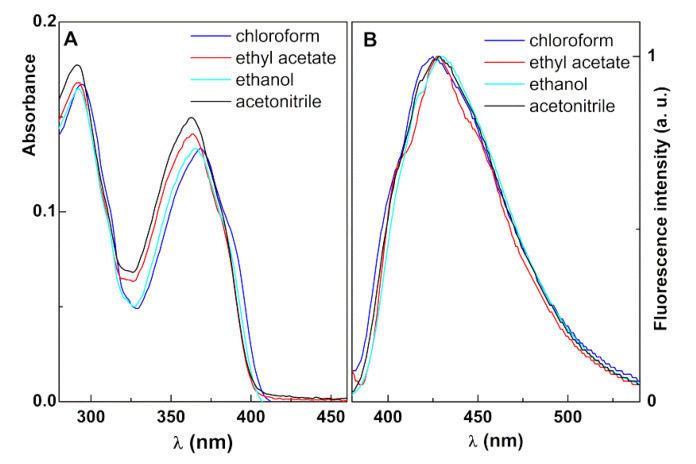
(**A**) Absorption spectra of 1 × 10^−5^ M solutions of compound 1 in ethyl acetate, acetonitrile, chloroform, and ethanol. (**B**) Normalized fluorescence spectra (*λ*_exc_ = 370 nm) of 1 × 10^−6^ M solutions of compound 1 in the same solvents.

**Figure 4:**
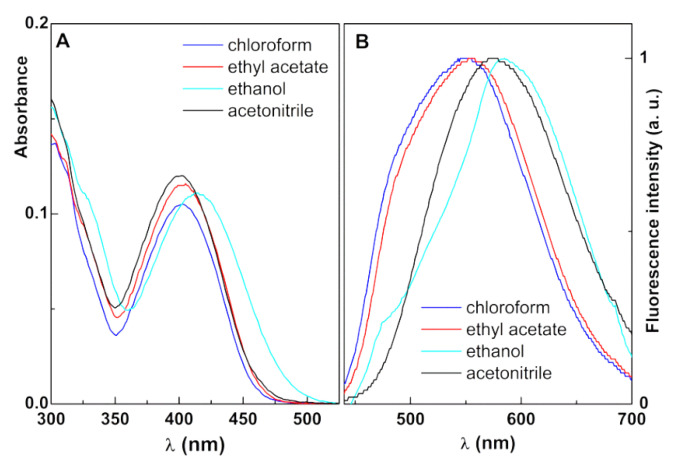
(**A**) Absorption spectra of 1 × 10^−5^ M solutions of compound 2 in ethyl acetate, acetonitrile, chloroform, and ethanol. (**B**) Normalized fluorescence spectra (*λ*_exc_ = 420 nm) of 1 × 10^−6^ M solutions of compound 2 in the same solvents.

The antitumor tricyclic lactones **1** and **2** are fluorescent compounds and this property can be used to monitor the compound location and behavior in the prepared nanosystems. First, UV-Vis absorption and fluorescence measurements were performed in several solvents of different polarity, to assess their potentialities to be used as intrinsic fluorescent probes when loaded in the developed nanosystems. The absorption and normalized fluorescence spectra are displayed in Figure 3 (for compound **1**) and Figure 4 (for compound **2**). The maximum absorption and emission wavelengths and molar absorption coefficients are shown in Table 2.

Both compounds exhibit fluorescence emissions in several solvents, but not in water, which is a common behavior in other thienopyridine derivatives previously studied [36,37,38]. Compound **2** exhibits a notable influence of solvent in the fluorescence emission, together with significant red shifts relative to compound **1**, which is a result of the influence of the electron-donating NH_2_ group. Both antitumor drugs present photophysical stability, as the absorption and emission of the two compounds are stable for several months (Figures S1 and S2 in Supplementary Materials).

### 3.2. Nanoparticles Synthesis and Characterization

#### 3.2.1. Synthesis of Manganese Ferrite/Gold Nanoparticles

Good-quality manganese ferrite nanoparticles were produced by co-precipitation, as already reported [39]. The gold nanoparticles were prepared in an organic phase using the Brust–Schiffrin method [29] and tetraoctylammonium bromide (TOAB) as Au(III) phase-transfer agent. Upon borohydride reduction, TOAB provides stabilization of the formed gold nanoparticles by capping them with octyl chains through the binding of (CH_3_(CH_2_)_7_)_4_N^+^Br^−^ ion pairs to the gold surface [40].

The decoration of manganese ferrite nanoparticles with gold was idealized through the use of cysteamine molecules (NH_2_CH_2_CH_2_SH) as bridges. CDI was chosen to enable the coupling of NH_2_ groups of cysteamine with hydroxyl (–OH) surface groups of manganese ferrite, with the remaining thiol (–SH) providing strong chemisorption sites for gold nanoparticles, thus resulting in gold-decorated magnetic manganese ferrite nanoparticles.

**Figure 5:**
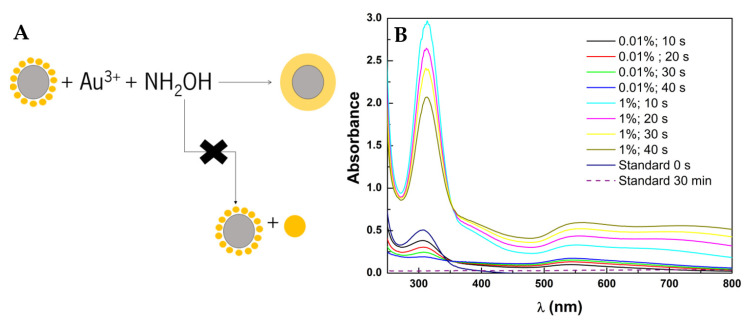
(**A**) Representation of the seeding method using hydroxylamine for the growth of a gold shell. (**B**) Absorption spectra of manganese ferrite/gold core/shell nanoparticles with increasing time, after the addition of hydroxylamine and chloroauric acid (0.01% and 1%).

Additional Au deposition on the decorating gold nanoparticles occurred via the “seeding” method using hydroxylamine as the reductant of Au^3+^ ions (Figure 5). The idea of that method consists of an enhanced Au^3+^ reduction reaction rate on the surface of pre-existent gold nanoparticles (seeds), so that the nucleation of new particles in the solution cannot compete with the growth of the seeds through the gold deposition process [30]. The coalescence of growing gold nanoparticles will eventually result in gold shell formation. Figure 5B evidences the growth of the gold shell with time and the addition of hydroxylamine.

**Figure 6:**
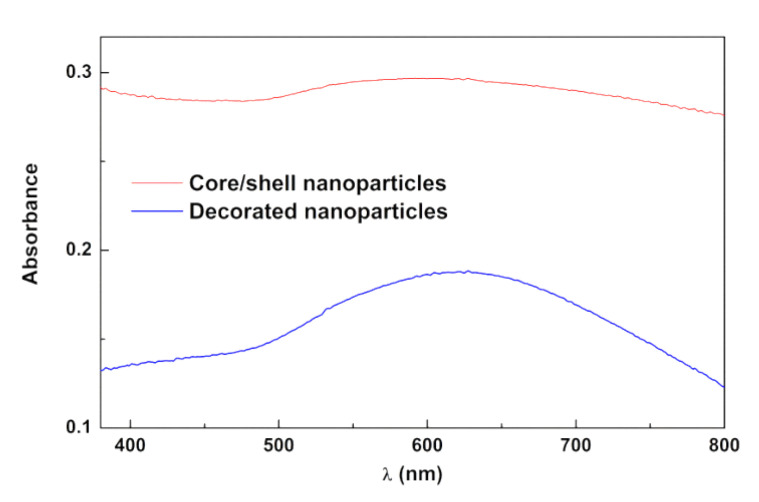
UV-Visible absorption spectra of aqueous dispersions of MnFe_2_O_4_ nanoparticles decorated with gold and MnFe_2_O_4_/Au core/shell nanoparticles.

The potential of manganese ferrite/gold core/shell nanoparticles (with a 5.85 nm gold shell) covered by lipid bilayers for phototherapy applications in cancer treatment was previously anticipated [41]. However, the magnetic properties of the nanoparticles were very poor, hindering the simultaneous application of magnetic hyperthermia, together with a limited photothermia effect [41]. More recently, nickel ferrite nanoparticles decorated with gold nanoparticles or with a gold shell, prepared by the same methodology used in this work, were revealed to be more promising for applications in thermotherapy [42]. However, toxicity issues arise when using nickel ferrite nanoparticles, also considering their easy degradation. So, manganese ferrite nanoparticles are preferred and have revealed no toxicity in primary cells when entrapped in liposomes [38]. Here, using the seeding method, manganese ferrite nanoparticles covered with a shell of gold and decorated with gold were prepared and covered with a liposome-like structure. The absorption spectra are shown in Figure 6. The absorption spectrum of manganese ferrite/gold core/shell nanoparticles exhibits a broader and red-shifted plasmon band relative to the decorated nanoparticles, with the plasmon band extending to 800 nm, allowing NIR irradiation.

The position of the plasmon absorption band of gold nanoshells varies with the shell thickness and the refraction index of both core and surrounding media [43]. Previous similar preparations of MnFe_2_O_4_ nanoparticles resulted in an average size of ~25 nm [39]. From published theoretical studies [44], it is possible to conclude that the gold resonance peak is predicted to occur at 595.6 nm for a gold shell of 5 nm over an air core of 25 nm in a water medium, while if the gold shell is 10 nm, it is expected to occur at 552.2 nm. The increase in the core refraction index results in a red shift of the plasmon resonance peak [43]. Thus, the absorption spectrum indicates a shell between 5 and 10 nm, while for the gold-decorated NPs, the spectrum is typical of clusters of gold spheres [45].

#### 3.2.2. XRD Analysis

**Figure 7:**
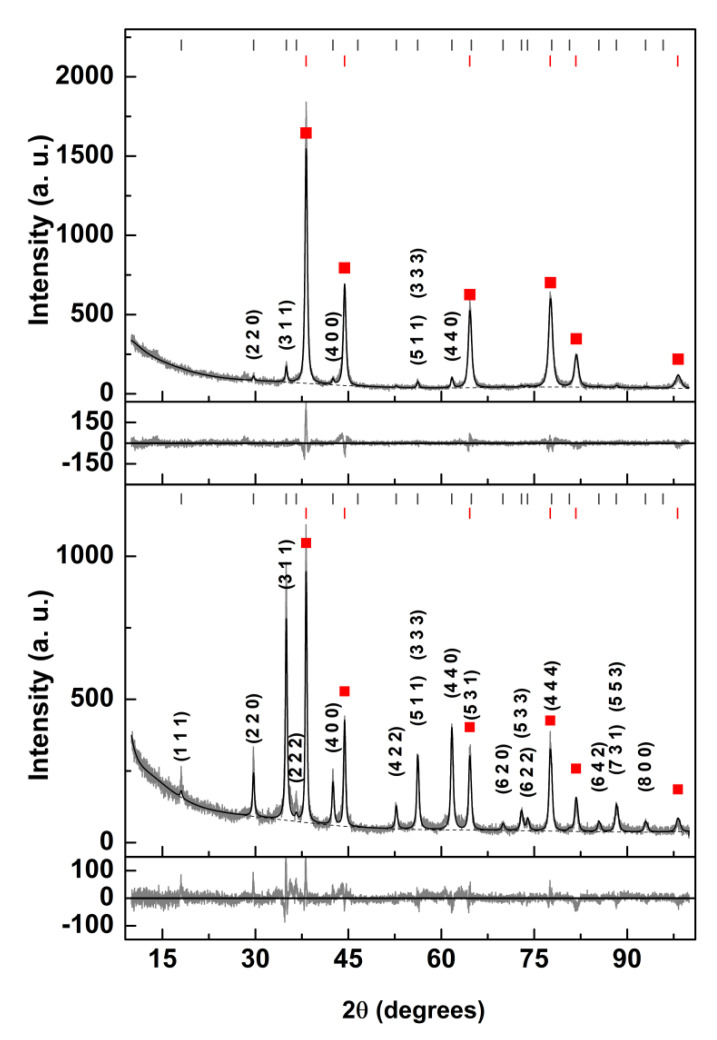
X-ray diffraction pattern of manganese ferrite/gold core/shell nanoparticles (above) and manganese ferrite decorated with gold nanoparticles (below). Gray lines: Experimental patterns; black lines: Fitted patterns; short-dashed lines: background. Miller indices of manganese ferrite are indicated in black; the gold peaks are marked as red squares.

XRD results of the prepared manganese ferrite conjugated with gold (Figure 7) confirm the crystallinity of the prepared nanoparticles. A Rietveld analysis was performed using BGMN [46], as implemented in Profex software [47]. The required structure files were obtained through the import process of CIF file nr. 9013035 for gold (space group Fm-3m) and CIF file nr. 2300618 for MnFe_2_O_4_ (space group Fd-3m). Diffraction peaks of gold are observed at 2θ = 38.2° (1 1 1), 44.4° (2 0 0), 64.6° (2 2 0), 77.6° (3 1 1), 81.7° (2 2 2), and 98.2° (4 0 0) and are marked with a filled red square in Figure 7, while for MnFe_2_O_4_, they occur at 2θ = 18.1° (1 1 1), 29.7° (2 2 0), 35.0° (3 1 1), 36.6° (2 2 2), 42.5° (4 0 0), 46.6° (3 3 1), 52.7° (4 2 2), 56.2° (5 1 1) and (3 3 3), 61.7° (4 4 0), 64.9° (5 3 1), 70.0° (6 2 0), 73.0° (5 3 3), 73.9° (6 2 2), 77.8° (4 4 4), 80.7° (7 1 1) and (5 5 1), 85.4° (6 4 2), 88.3° (7 3 1) and (5 5 3), and 93.0° (8 0 0). All diffraction peaks are identified, so there are no other crystallographic phases present in the sample. Good fits were obtained, and the main results obtained by the Rietveld analysis are shown in Table 3.

A huge increase in the gold phase diffraction peaks’ intensity occurs as a result of the seeding process (Figure 7). This confirms the predictable increase in gold content that would result from the growth of the spherical gold NPs decorating the MnFe_2_O_4_ surface expectedly into a gold shell. 

Considering that, for core-shell nanoparticles, a given X-ray interacts with the gold phase two times, 96% gold content would be expected (56.7 nm overall size; 27.9/2 = 14 nm shell thickness). A shell thickness of 5 nm (38.8 nm overall size) is more compatible with the observed absorption spectra and corresponds to a weight percentage of 84.7%. This allows us to estimate a coating efficiency of 86%. The size obtained from the broadening of gold diffraction peaks has been found to be unreliable [41,49].

For the gold-decorated NPs, the 41.9 nm size, obtained for the gold phase from the Rietveld analysis, is not compatible with the 5–7 nm value reported for gold spherical nanoparticles synthesized with a similar method to the one used in this work [50]. Even if the size of gold-decorated NPs is 41.9/2 nm, the average number of spherical gold NPs per decorated MnFe_2_O_4_ nanoparticle is estimated to be 0.078, from the determined gold weight percentage of 12.8%. Considering the 5 to 7 nm reported size range, that average number is estimated to be, respectively, from 5.8 to 2.1. This range is much more reasonable, indicating that the size of the gold phase determined from XRD analysis is affected by unknown effects and/or experimental artifacts. 

#### 3.2.3. Transmission Electron Microscopy (TEM)

TEM images of manganese ferrite nanoparticles shelled with gold or decorated with gold nanoparticles are shown, respectively, in Figure S3A,C in Supplementary Materials. The decorated nanoparticles image shows distinct features (Figure S3E in Supplementary Materials), that consist of small particles arranged in a pattern, in areas with underlying intensity clearly different from the image background. These can be interpreted as ferrite nanoparticles with small gold nanoparticles above their surface. The histograms in Figure S3B,D,F (see Supplementary Materials) were obtained by manually outlining the particles in, respectively, Figure S3A,C,E, using the elliptical selection tool of ImageJ and considering its size as the diameter of a circle with an equivalent area. Gaussian distribution fits to the histograms allowed size estimations of 30 ± 4 nm for the core/shell nanoparticles, 46 ± 9 nm in the case of the decorated nanoparticles, and 10 ± 2 nm for the small particles observed in Figure S3E. These size estimations are compatible with the discussion of the XRD results. Further evidence is shown in Figure S4 in Supplementary Materials, using an SEM microscope. STEM images of core/shell nanoparticles (in Lacey carbon film-coated EM grids) show isolated particles with sizes within the histogram in Figure S3B. The EDS spectrum in Figure S4C confirms the presence of gold and atomic percentages that are compatible with the existence of MnFe_2_O_4_. The topography of the NPs’ surface exhibited in Figure S4D, through the detection of secondary electrons (SE) by the TLD detector, shows either particles covered by a very bright homogeneous layer (marked with a thin arrow) or particles with bright spots (marked with a thick arrow). The latter could be NPs decorated with gold that, through the seed-mediated growth process, have not fully evolved to a continuous gold layer that corresponds to the former.

#### 3.2.4. Magnetic Properties

**Figure 8:**
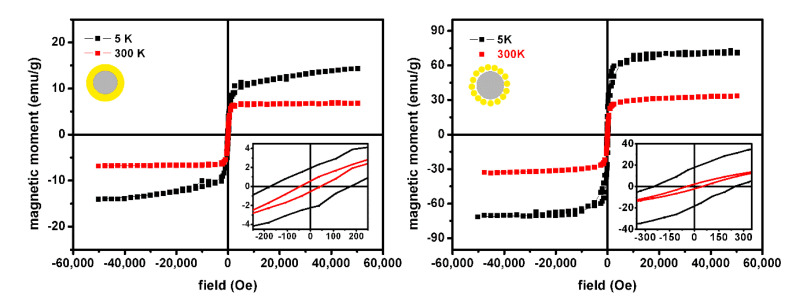
Magnetization hysteresis loops of MnFe_2_O_4_/Au core/shell nanoparticles (left) and MnFe_2_O_4_ NPs decorated with Au (right) at 5 K and 300 K. Insets: Enlargement of the loops in the low field region.

Manganese ferrite nanoparticles are soft magnetic materials with interesting magnetic properties for biomedical applications, yet such properties strongly depend on the surface, due to increased effects arising from the size decrease. Surface functionalization with gold can lead to increased surface effects of the magnetic core. The surface magnetic moments of the core can be disordered by the interaction with gold electrons leading to a larger spin canting resulting in an overall decrease in magnetic properties. Gold has a density four times larger than manganese ferrite and is diamagnetic in the bulk state. However, a ferromagnetic behavior is expected for very small-sized nanoparticles (4–5 nm) [51]. Therefore, in this work, the magnetic contribution of the overall structure of the magnetic/plasmonic nanoparticles strongly depends on the gold shell or NP size on the surface of the magnetic core. The magnetic properties of the synthetized magnetic/plasmonic nanoparticles were characterized by measuring the magnetic hysteresis loop (Figure 8), which represents the magnetization (M) as a function of the applied magnetic field (H)). Typically, manganese ferrite nanoparticles have a partially inverse spinel structure, with about 80% of Mn^2+^ ions located at tetrahedral sites and only 20% at octahedral sites [48]. Knowing that Mn^2+^ and Fe^3+^ ions contribute to large magnetic moments (5 µ_B_) and that the structural formula for ion distribution is represented by (Mn_1−δ_Fe_δ_↑)[Fe_2−δ_Mn_δ_↓]O_4_, the expected net magnetic moment of manganese ferrite NPs is 2.0 µ_B_ per MnFe_2_O_4_ (for δ = 0.20) [52].

Figure 8 displays the hysteresis loops of the magnetic/plasmonic samples measured at 300 K and 5 K, the low field region being shown at insets. The saturation magnetization of magnetic/plasmonic NPs at 300 K exhibits lower values than those measured at 5 K, due to the thermal fluctuations of nanoparticle magnetic moments at higher temperatures. At 5K, both samples show larger hysteresis with higher values of remnant magnetization and the coercive field, presenting ferromagnetic behavior with a corresponding magnetic squareness ratio above 0.1 (Table 4).

At room temperature, synthetized nanoparticles have shown higher hysteresis compared to MnFe_2_O_4_ NPs obtained in previous research [39], yet the magnetic/plasmonic structures have shown superparamagnetic behavior. Both structures (decorated or core/shell) have shown a magnetic squareness ratio below 0.1 (Table 4), indicating that more than 90% of the induced magnetization was lost upon the removal of the external magnetic field [52], hence being promising for biomedical applications. Comparing the saturation magnetization value, at room temperature, of the MnFe_2_O_4_ NPs previously obtained [39] with the magnetic/plasmonic ones, it is possible to observe a slight decrease for the decorated structure, contrasting with a strong decrease for that of the core/shell (Table 4) as was predicted considering the diamagnetic nature of the gold shell. The small decrease in saturation magnetization for the nanoparticles decorated with gold can be justified by small and/or incomplete coverage of the magnetic core with gold, as observed for nickel ferrite [42]. In addition, this higher value of magnetization of gold-seeded manganese ferrite nanoparticles may also result from an aggregation process that occurs during the seeding process. For the core/shell nanoparticles, a higher *M*_s_ value was obtained than for similar nanoparticles prepared by another method [41].

Considering a well-ordered core of MnFe_2_O_4_ covered by a non-magnetic gold shell (“dead layer”), it was possible to estimate the thickness of the gold shell using the magnetic hysteresis cycle, by taking the relation between the saturation magnetization, *M_s_*, to the particle diameter (*D*) and the saturation magnetization of the core ($M_{s0\;}$). The thickness *δ* can be calculated through Equation (3) [52],

(3) $$M_{s}=M_{s0\;}\left(1-\frac{6\delta }{D}\right)$$

The saturation magnetization depends on the particle size. Chen et al. [53] reported a saturation magnetization of 58 emu/g for MnFe_2_O_4_ nanoparticles of 13.3 nm prepared by co-precipitation. With this value, a gold shell with a thickness of 4.23 nm was estimated for particles with a diameter of 28.8 nm (from XRD) and *M_s_* = 6.89 emu/g (from the hysteresis cycle).

The magnetic properties of manganese ferrite nanoparticles decorated with gold nanoparticles and core/shell nanostructures show enhanced saturation magnetization relative to that previously determined for NiFe_2_O_4_/Au core/shell NPs or NiFe_2_O_4_ decorated with Au [42].

### 3.3. Drug-Loaded Magnetoliposomes

**Figure 9:**
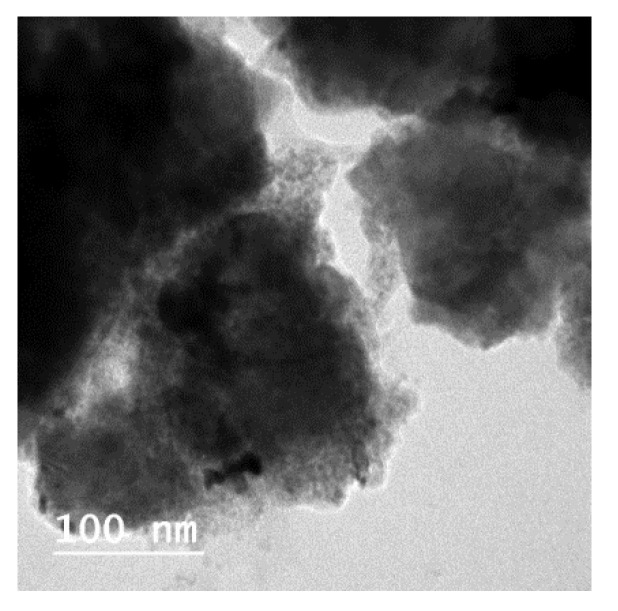
TEM image of magnetoliposomes containing magnetic nanoparticles decorated with gold.

Magnetoliposome-type structures were prepared, possessing an octadecylamine (ODA) inner layer and a DPPC lipid outer layer, as previously described [42]. The nanosystems were characterized by DLS in PBS buffer (Table 5), making it possible to observe hydrodynamic diameters below 200 nm (that are maintained after 7 days), as is requested for an enhanced EPR (Enhanced Permeability and Retention) effect, which allows the extravasation of liposomes and passive targeting [54,55]. TEM images of these nanosystems evidence mainly spherical structures with sizes around 100 nm, although aggregated (Figure 9), in agreement with DLS data. The aggregation observed in TEM images is due to the technique employed, which requires a dry film of the sample in a solid grid and the application of a vacuum. This procedure causes the aggregation of the nanostructures in the grid and, consequently, in TEM images.

**Figure 10:**
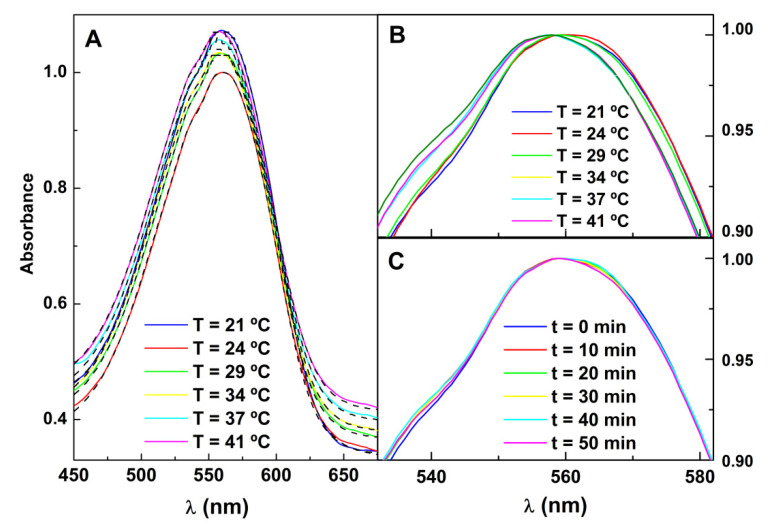
(**A**) Variation of absorption spectra of Nile Red in magnetoliposomes containing core/shell nanoparticles, at different temperatures and fitting to the model (dashed curves). (**B**) Expansion of the normalized spectra of Figure A in the maxima region. (**C**) Expansion of the normalized spectra in the maxima region at several times of irradiation.

**Figure 11:**
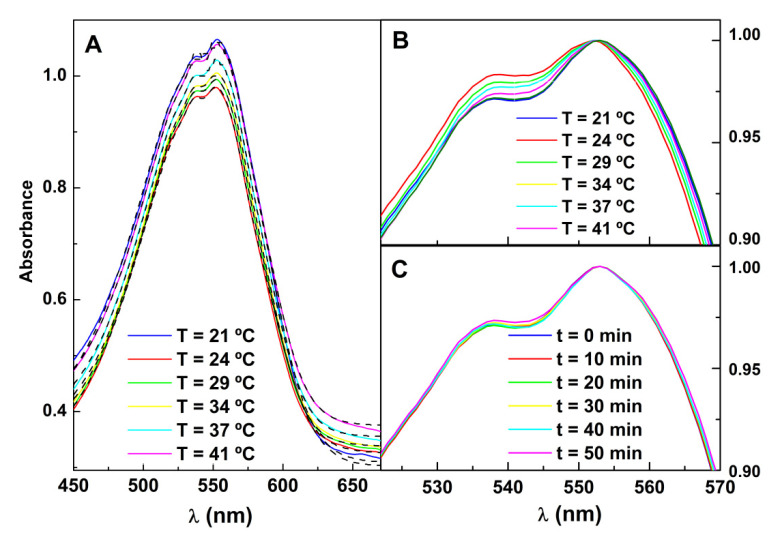
(**A**) Variation of absorption spectra of Nile Red in magnetoliposomes containing decorated nanoparticles, at different temperatures and fitting to the model (dashed curves). (**B**) Expansion of the normalized spectra of Figure A in the maxima region. (**C**) Expansion of the normalized spectra in the maxima region at several times of irradiation.

The ability of magnetoliposomes to act as photothermia agents was measured through variations in the absorption spectrum of the lipid probe Nile Red. This molecule was previously shown to have thermochromic behavior [56]. Figure 10A and Figure 11A exhibit absorption spectra of Nile Red when incorporated in magnetoliposomes containing, respectively, core/shell manganese ferrite/gold NPs or gold-decorated manganese ferrite NPs, as a function of the medium temperature. These absorption spectra were acquired in a home-made experimental setup that is represented in Scheme 1, using a water bath as a temperature controller. A magnification of the absorption changes is shown in Figure 10B and Figure 11B. For each system, in order to model the variations, the absorption spectra were globally fitted with a sum of eight Gaussian functions, constrained to a sequence of spectral zones and controlled halfwidth (see Supplementary Material and Table S1). The maximum wavelength and halfwidth of each Gaussian function are global parameters of the fit, which are the same for all the temperatures. The weight of each Gaussian function was allowed to vary with temperature, with the three parameters that define such variation also being global parameters for all the temperatures. Additionally, a dispersion contribution with Rayleigh-like behavior was considered for each spectrum. A successful fit was obtained, as can be observed in Figure 10A and Figure 11A. The set of global parameters was then used to fit the absorption spectra that result from the irradiation of the same samples (Figure 10C and Figure 11C), using the same experimental setup (Scheme 1). The temperature at each irradiation time is now a local parameter of the global fitting procedure. The obtained local temperatures that result from red light (λ > 600 nm) irradiation of the magneto/plasmonic liposomes are shown in Table 6.

From Table 6, it is possible to conclude that the core/shell nanoparticles are much more effective in increasing the local temperature than the decorated ones, allowing a local increase of 3 °C. This value is an underestimation, as during the time the absorption spectra were measured, it was necessary to block the irradiation light. This results in heat diffusion into the solvent during the measurement time, as the plasmonic heating sources are no longer active.

The potential of magnetoliposomes as nanocarriers for antitumor compounds **1** and **2** was assessed. Using the intrinsic fluorescence of the new tricyclic lactones, it is possible to infer the main location of compounds in magnetoliposomes through fluorescence anisotropy measurements. First, considering that compounds are not emissive in aqueous media, the observed fluorescence in magnetoliposomes points to a location in the surfactant/lipid bilayer. Moreover, the fluorescence anisotropy values (r = 0.071 for compound **1** and r = 0.115 for compound **2**) indicate a deep location in the bilayer, in the fluid region of membrane interior, as anisotropy values are moderate to low.

The drug encapsulation efficiencies were determined for both compounds (Table 7) in magnetoliposomes containing the two types of nanoparticles (decorated or core/shell), also using fluorescence emission. The encapsulation efficiencies are very high, confirming the affinity of both compounds by magnetoliposomes. 

**Figure 12:**
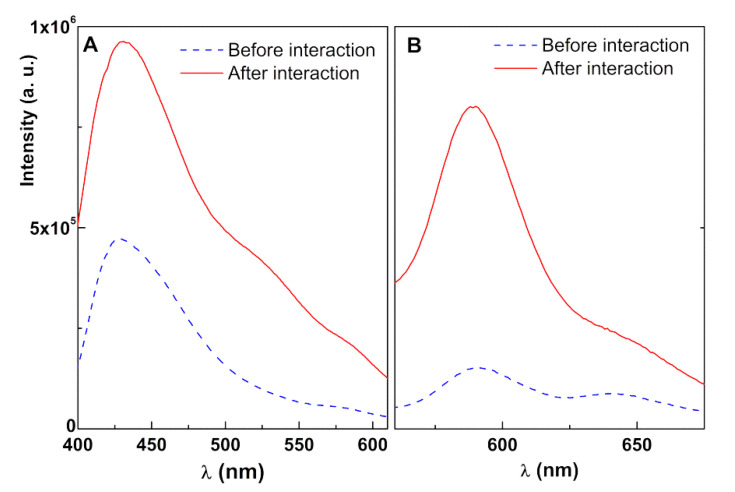
Fluorescence spectra of (**A**) compound **1** (*λ*_exc_ = 370 nm) and (**B**) compound **2** (*λ*_exc_ = 420 nm) in magnetoliposomes before and after interaction with GUVs.

The interaction with biomembrane models was assessed to infer whether the antitumor compounds can be released from the nanocarriers by fusion with cell membranes. As biomembrane models, Giant Unilamellar Vesicles (GUVs) were used [31,32]. The fusogenic capability was investigated by fluorescence emission. After the interaction of the drug-loaded magnetoliposomes with GUVs, a notable fluorescence unquenching (increase in fluorescence emission) is detected for both drugs (Figure 12). This is a result of the increase in distance between the drugs and the magnetic/plasmonic nanoparticles. The latter, when in close proximity to the drugs, absorbs part of the excitation light, producing a decrease in fluorescence emissions of the compounds. Compound **2** seems to present two different locations in the lipid nanosystems, as two bands are present in the fluorescence spectrum (Figure 12B). A small band with a maximum size of approximately 640 nm may indicate a more hydrated environment (near the lipid head groups).

### 3.4. Cytotoxicity Assays in Cell Lines

**Figure 13:**
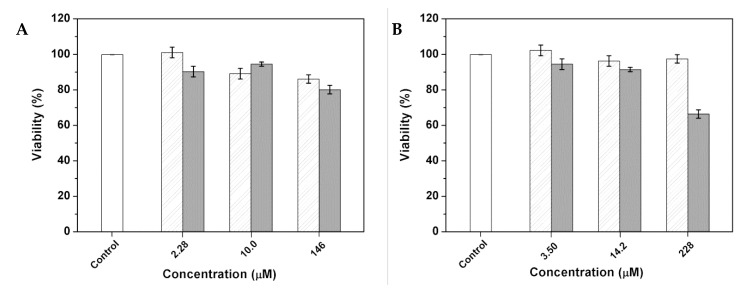
Viability of PLP2 cells exposed to drug-loaded nanocarriers based on MnFe_2_O_4_/Au core/shell NPs (dashed column) and nanocarriers based on MnFe_2_O_4_ decorated with Au (grey column), under irradiation. (**A**) Compound **1**; (**B**) Compound **2**.

The biological activity of the developed nanosystem was evaluated in normal (non-tumor) and human tumor cell lines. The toxicity studies of the drug-loaded nanocarriers towards non-tumor cells, using porcine liver primary cells (PLP2), were performed under irradiation (Figure 13). No appreciable loss of viability was observed, except for very high concentrations of compound **2** in one of the nanocarriers. These results evidence the biocompatibility of the drug-loaded nanocarriers under irradiation in non-tumor cells. Hence, it must be emphasized that the local rise in temperature seems to be insufficient to kill non-tumor cells. 

**Figure 14:**
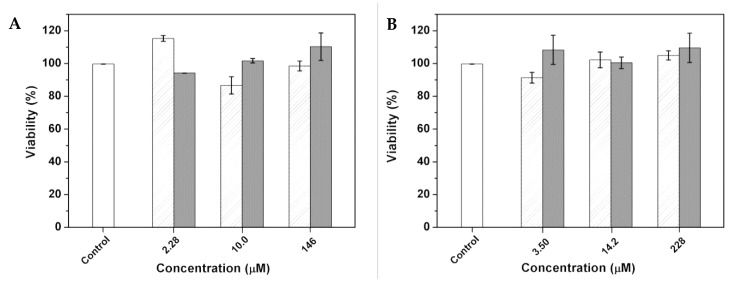
Viability of NCI-H460 tumor cells exposed to drug-loaded nanocarriers containing MnFe_2_O_4_/Au core/shell NPs (dashed column) and containing MnFe_2_O_4_ decorated with Au NPs (grey column), without irradiation. (**A**) Compound **1**; (**B**) Compound **2**.

Among the deadliest cancer types are, in descending order, the cancers of the lungs, prostate, and colorectal cancer in men and the lungs, breast, and colorectal cancer in women [1]. These facts led us to investigate the growth inhibitory activity of the drug-loaded nanocarriers in human NCI-H460 cell lines (non-small-cell lung cancer). As previously mentioned, both compounds exhibit promising antitumor activity for this type of cancer cells (Table 1). The assays in the NCI-H460 cell line were carried out in the presence and absence of irradiation to determine the effective photothermia capability of the developed nanosystems. In the absence of irradiation, no loss of viability was observed in tumor cell lines (Figure 14), even at very high compound concentrations, showing that the antitumor compounds are not released in the absence of the local temperature rise promoted by the plasmonic effect of gold. On the contrary, when irradiation with light was applied, notable growth-inhibitory activity was observed in tumor cells for the nanocarriers containing the core/shell nanoparticles. The GI_50_ values (the concentration of which inhibited 50% of the cell growth) are very low, with values around 100 nM (Table 8). The GI_50_ is slightly lower for the more active antitumor compound (compound **1**). This clearly demonstrates the synergistic effect between photothermia and drug cytotoxicity to tumor cells, enhancing the antitumor activity by the local rise in temperature promoted by the plasmonic effect of gold. On the other hand, for the nanosystems containing the magnetic nanoparticles decorated with gold, the growth inhibition of 50% of tumor cells was not attained. Moreover, the decorated nanoparticles seem not to have enough quantity of gold for photothermia-triggered drug release. This is in accordance with the previously reported incomplete covering of nickel ferrite nanoparticles with gold in the decorated magnetic/plasmonic NPs [42].

Overall, these results demonstrate that the drug-loaded magnetoliposomes with core-shell nanoparticles are suitable for a light-triggered release in non-small cell lung cancer, because (i) no cell viability loss was observed in normal cells (under irradiation), and (ii) in NCI-H460 tumor cells, the nanosystem only showed growth inhibitory activity in the presence of irradiation. These results anticipate the promising application of these nanosystems in non-small-cell lung cancer treatment, using irradiation as a trigger for drug release. 

## 4. Conclusions 

Nanoparticles consisting of MnFe_2_O_4_ magnetic nanoparticles either decorated with plasmonic gold nanoparticles or covered by a plasmonic gold shell were successfully obtained. A surfactant/lipid (ODA/DPPC) bilayer was used to wrap up these nanoparticles, leading to the synthesis of a liposome-like structure. The nanosystems were loaded with two new potential antitumor tricyclic lactones. The drug-loaded nanosystems exhibited hydrodynamic sizes below 200 nm, high encapsulation efficiencies, and a fusogenic capability with model membranes. The drug-loaded magnetoliposomes containing MnFe_2_O_4_/gold core/shell nanoparticles were shown to be promising in future applications in dual cancer therapy, combining photothermia and chemotherapy.

## Figures and Tables

**Table 1:** Growth inhibitory concentrations (GI_50_) of compounds **1** and **2** in human tumor cell lines.

GI_50_ (μM) [28]	Compound 1	Compound 2
HCT-15	7.4 ± 1.1	12.6 ± 2.9
NCI-H460	7.3 ± 0.1	11.4 ± 0.9

**Table 2:** Maximum absorption (λ_abs_) and emission (λ_em_) wavelengths and molar absorption coefficients (ε) for compounds **1** and **2** in several solvents.

Solvent	λ_abs_/nm (ε/10^4^ M^−1^ cm^−1^)		λ_em_/nm	
	Compound 1	Compound 2	Compound 1	Compound 2
Chloroform	369 (1.33)	403 (1.05)	425	523
Ethyl acetate	364 (1.41)	405 (1.16)	427	532
Ethanol	365 (1.33)	415 (1.10)	430	581
Acetonitrile	363 (1.50)	403 (1.21)	428	568

**Table 3:** Calculated R_P_ and χ^2^ parameters, phase sizes, and percentages obtained by Rietveld refinement of X-ray diffraction patterns of Au@MnFe_2_O_4_ (gold-decorated nanoparticles) and MnFe_2_O_4_/Au (core/shell nanoparticles).

Sample	O_x,y,z_ (*)	*i* (**)	Phase Size (nm) Lattice Constant (nm)		Au (wt%)	R_P_	χ^2^
			MnFe_2_O_4_	Au			
Au@MnFe_2_O_4_	0.3865	0.2 (^+^)	26.7 0.8498	41.9 0.4078	12.8	10.5	1.96
MnFe_2_O_4_/Au	0.3885	0.2 (^+^)	28.8 0.8497	27.9 0.4078(+)	74.7	9.4	1.62

(*) Value of O_x,y,z_ in CIF file 2300618 is 0.25053 (**) Degree of inversion (^+^) fixed to value in reference [48].

**Table 4:** Coercive field (*H*_c_), remnant magnetization (*M*_r_), saturation magnetization (*M*_s_), and magnetic squareness ratio (*M*_r_/*M*_s_) of MnFe_2_O_4_/Au core/shell nanoparticles and MnFe_2_O_4_ NPs decorated with Au, at 5 K and 300 K. The magnetic properties of MnFe_2_O_4_ nanoparticles are also shown for comparison [39].

	T (K)	*H*_c_ (Oe)	*M*_r_ (emu/g)	*M*_s_ (emu/g)	*M*_r_/*M*_s_
MnFe_2_O_4_ [39]	300	6.30	0.58	36.00	0.02
MnFe_2_O_4_ NPs decorated with Au NPs	5	247.47	17.68	71.02	0.25
	300	46.23	2.17	33.66	0.06
MnFe_2_O_4_/Au core/shell NPs	5	173.82	1.93	14.43	0.13
	300	45.95	0.55	6.89	0.08

**Table 5:** Hydrodynamic size and polydispersity (PdI) of magnetoliposomes in PBS buffer determined by DLS. (SD: standard deviation).

Type of NPs	Size ± SD (nm)		PdI ± SD	
	After Preparation	After 7 Days	After Preparation	After 7 Days
Magnetoliposomes with decorated NPs	124 ± 28	136 ± 34	0.28 ± 0.08	0.32 ± 0.062
Magnetoliposomes with core-shell NPs	132 ± 21	147 ± 28	0.23 ± 0.04	0.26 ± 0.05

**Table 6:** Local temperature as determined by Nile Red absorption changes.

Irradiation Time (min).	Magnetoliposomes with Decorated NPs	Magnetoliposomes with Core/Shell NPs
0	21 °C	21 °C
10	21.6 °C	21.8 °C
20	22.5 °C	23.1 °C
30	22.8 °C	23.8 °C
40	22.9 °C	23.9 °C
50	22.9 °C	24.0 °C

**Table 7:** Encapsulation efficiencies (EE%) of antitumor compounds **1** and **2**. SD is the standard deviation of three independent assays.

System	EE% ± SD%	
	Compound 1	Compound 2
Magnetoliposomes with core-shell NPs	99.3 ± 0.8	99.9 ± 0.5
Magnetoliposomes with decorated NPs	98.5 ± 1.3	99.8 ± 0.6

**Table 8:** Growth inhibitory concentrations (GI_50_) in NCI-H460 lung cancer cell line. The results are from three independent experiments (performed in triplicate) and are expressed as mean ± standard deviation (SD).

System	GI_50_ ± SD (μM)	
	Compound 1	Compound 2
Drug-loaded magnetoliposomes with decorated NPs	---	---
Drug-loaded magnetoliposomes with core-shell NPs	0.11 ± 0.02	0.12 ± 0.002

## Data Availability

Not applicable.

## Supplementary Materials

The following are available online at https://www.mdpi.com/article/10.3390/pharmaceutics13111905/s1: Modeling of Nile Red absorption spectra, Table S1. Gaussian functions parameters. Photophysical stability of compound solutions, Figure S1. Fluorescence emission spectra of compound 1 in chloroform (A) and ethanol (B) (as examples), immediately after solution preparation and after 1 and 3 months of storage; Figure S2. Fluorescence emission spectra of compound 2 in ethyl acetate (A) and acetonitrile (B) (as examples), immediately after solution preparation and after 1 and 3 months of storage. TEM/STEM images and EDS analysis, Figure S3. (A) Transmission Electron Microscopy image of core/shell nanoparticles and (B) corresponding histogram. (C) Transmission Electron Microscopy image of decorated nanoparticles and (D) corresponding histogram. (E) Expansion of image C (white square) and (F) corresponding histogram; Figure S4. (A) and (B) Scanning electron microscopy images in transmission mode (STEM) of core/shell nanoparticles. (C) EDS spectrum of region Z1 (red square) from figure (A). (D) TLD image.
