# Supplementary material for: Magnetoliposomes Based on Magnetic/Plasmonic Nanoparticles Loaded with Tricyclic Lactones for Combined Cancer Therapy
Source: Pharmaceutics. 2021 Nov 10;13(11):1905. doi: 10.3390/pharmaceutics13111905 (PMC8625448; doi:10.3390/pharmaceutics13111905)
Supplement: Supplementary file 1 [file pharmaceutics-13-01905-s001.zip › pharmaceutics-1346075-supplementary.pdf]

pharmaceutics

Supplementary Material

Magnetoliposomes Based on Magnetic/Plasmonic Nanoparticles Loaded with Tricyclic Lactones for Combined Cancer Therapy

Irina S. R. Rio [1], Ana Rita O. Rodrigues [1], Juliana M. Rodrigues [2], Maria-João R. P. Queiroz [2], R. C. Calhelha [3], Isabel C. F. R. Ferreira [3], Bernardo G. Almeida [1], Ana Pires [4], André M. Pereira [4], João P. Araújo [4], Elisabete M. S. Castanheira [1,*] and Paulo J. G. Coutinho [1,*]

[1] Centre of Physics of Minho and Porto Universities (CF-UM-UP), University of Minho, Campus de Gualtar, 4710-057 Braga, Portugal; id9261@alunos.uminho.pt (I.S.R.R.); ritarodrigues@fisica.uminho.pt (A.R.O.R.); bernardo@fisica.uminho.pt (B.G.A.)
[2] Centre of Chemistry (CQUM), University of Minho, Campus de Gualtar, 4710-057 Braga, Portugal; juliana.mourarodrigues@gmail.com (J.M.R.); mjrpq@quimica.uminho.pt (M.-J.R.P.Q.)
[3] Mountain Research Centre, Polytechnic Institute of Bragança, Campus de Santa Apolónia, 5300-253 Bragança, Portugal; calhelha@ipb.pt (R.C.C.); iferreira@ipb.pt (I.C.F.R.F.)
[4] IFIMUP—Instituto de Física dos Materiais, Universidade do Porto, R. Campo Alegre, 4169-007 Porto, Portugal; ana.pires@fc.up.pt (A.P.); ampereira@fc.up.pt (A.M.P.); jearaujo@fc.up.pt (J.P.A.)
* Correspondence: ecoutinho@fisica.uminho.pt (E.M.S.C.); pcoutinho@fisica.uminho.pt (P.J.G.C.); Tel.: +351-253-604-321 (E.M.S.C. & P.J.G.C.)

Modeling of Nile Red absorption spectra

A set of absorption spectra of the dye Nile Red, at several temperatures, were globally fitted to a sum of eight Gaussian functions and a dispersive background, as follows:

$$g_j(\lambda) = \frac{1}{\sigma_j\sqrt{2\pi}} e^{-\frac{1}{2}\left(\frac{\lambda-\lambda_{max,i}}{\sigma_j}\right)^2} \tag{1}$$

$$Abs(\lambda, T_i) = A_{disp}(\lambda, T_i) + A(T_i)\sum_{j=1}^{8} f_j g_j(\lambda) \tag{2}$$

$$A_{disp}(\lambda, T_i) = A_{offset}(T_i) + \frac{A_d(T_i)}{(\lambda/100)^4} \tag{3}$$

$$f_1(T_i) = \sin^2\left(a_1 + b_1 \times \left(\frac{T_i - T_0}{T_0}\right) + c_1 \times \left(\frac{T_i - T_0}{T_0}\right)^2\right) \tag{4}$$

$T_0 = 21$ °C

$$f_j(T_i) = \left(1 - \sum_{k=1}^{j-1} f_k\right)\sin^2\left(a_j + b_j \times \left(\frac{T_i - T_0}{T_0}\right) + c_j \times \left(\frac{T_i - T_0}{T_0}\right)^2\right) \tag{5}$$

$j = 2, 3, \ldots, 7$

$$f_8(T_i) = \left(1 - \sum_{k=1}^{j-1} f_k\right) \tag{6}$$

In order to control the fitting procedure, the parameters of the Gaussian functions were constrained as follows:

$$\lambda_{max,j} = \left(\lambda_{max,j}\right)_{min} + \left[\left(\lambda_{max,j}\right)_{max} - \left(\lambda_{max,j}\right)_{min}\right] \sin^2(m_j) \quad (7)$$

$$\sigma_j = \left(\sigma_j\right)_{min} + \left[\left(\sigma_j\right)_{max} - \left(\sigma_j\right)_{min}\right] \sin^2(s_j) \quad (8)$$

Table S1. Gaussian functions parameters.

j	1	2	3	4	5	6	7	8
$\left(\lambda_{max,j}\right)_{min}$	510	520	530	540	550	560	575	590
$\left(\lambda_{max,j}\right)_{max}$	520	530	540	550	560	575	590	630
$\left(\sigma_j\right)_{min}$	5	5	5	5	5	5	5	5
$\left(\sigma_j\right)_{max}$	50	10	10	40	10	40	50	50

The global fitting procedure for each set of absorption spectra with varying temperature consists on minimizing the following quadratic error sum:

$$ErrorSum = \sum_{i=1}^{n_T} \sum_{l=1}^{n_\lambda} w_l \left(Abs(\lambda_l, T_i) - Abs_{experimental}(\lambda_l, T_i)\right)^2 \quad (9)$$

where w_l is a weight at each wavelength of absorption. It was taken as 1, except in the region 520–570 nm, where a value of 50 was used. This procedure ensures a good fit where the spectrum has more features.

The parameters that are varied in order to reach a minimum in the *ErrorSum* are A_{offset} and A_d for each absorption spectrum; a_1 to a_7; b_1 to b_7; c_1 to c_7; m_1 to m_8 and s_1 to s_8.

We used six temperatures, so that each absorption spectrum is fitted with an average of 2 + (3 × 7 + 2 × 8)/6 = 8.17 parameters. This is equivalent to two parameters for the dispersive background and between 2 and 3 Gaussian functions (5 to 8 parameters).

For the case of the fitting of spectra obtained as a function of irradiation time, the fitted parameters are A_{offset}, A_d and T_i for each irradiation time.

Photophysical stability of compound solutions

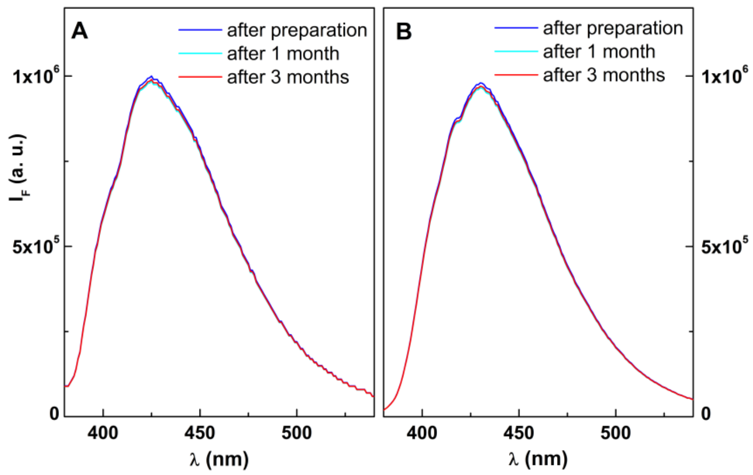

Figure S1. Fluorescence emission spectra of compound **1** in chloroform (**A**) and ethanol (**B**) (as examples), immediately after solution preparation and after 1 and 3 months of storage.

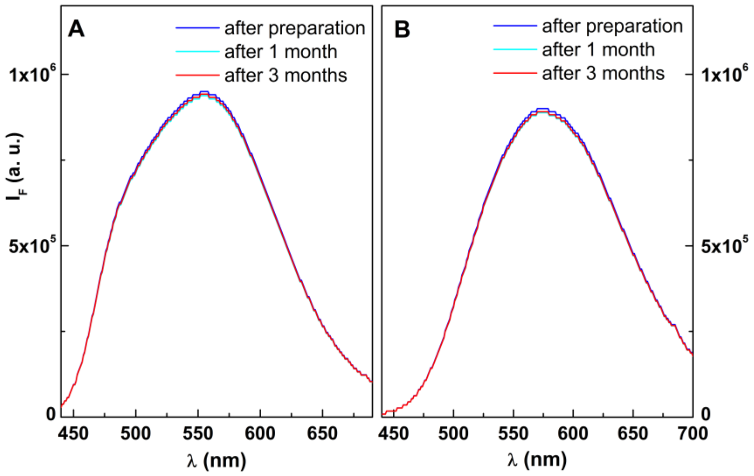

Figure S2. Fluorescence emission spectra of compound **2** in ethyl acetate (**A**) and acetonitrile (**B**) (as examples), immediately after solution preparation and after 1 and 3 months of storage.

TEM/STEM images and EDS analysis

Transmission electron microscopy (TEM)

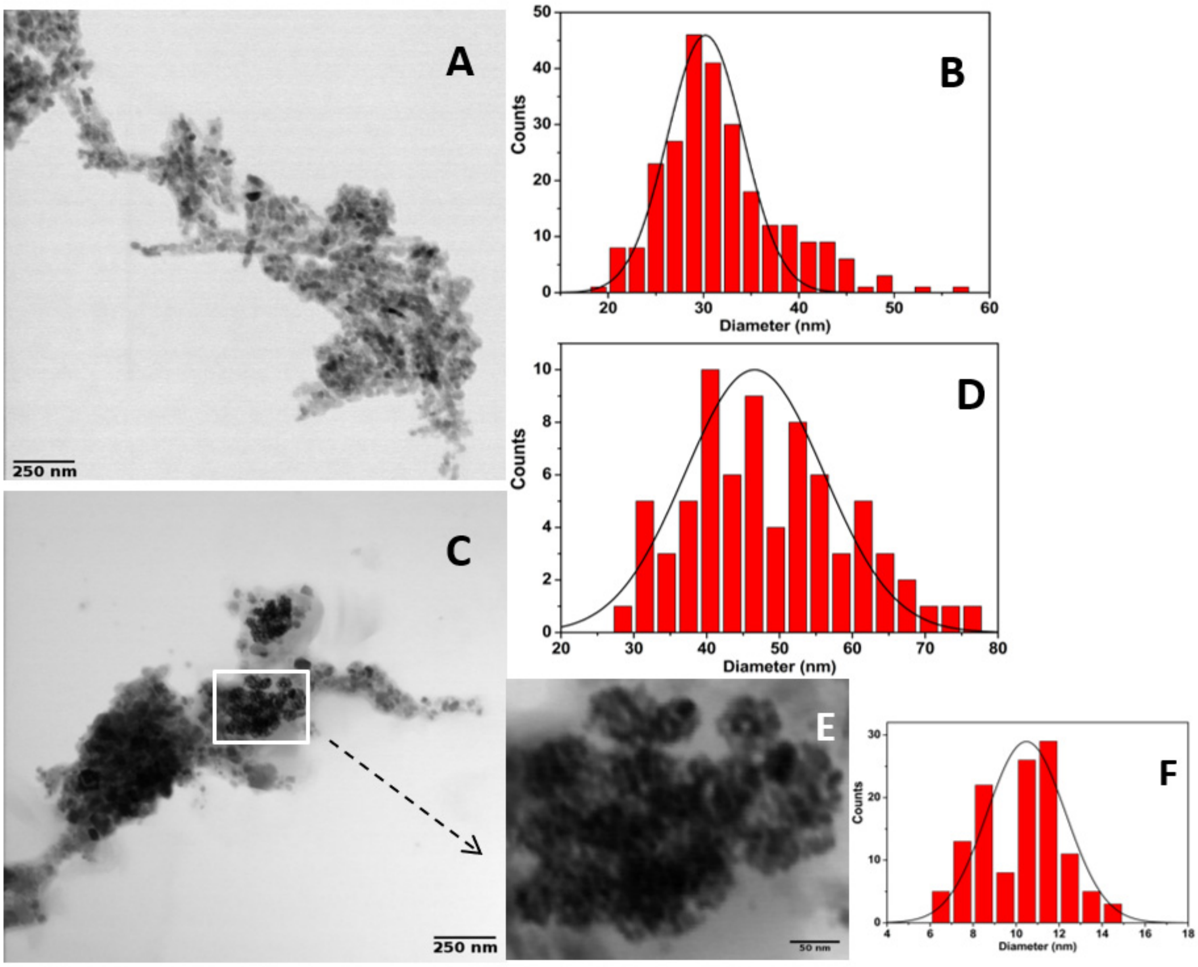

Figure S3. (**A**) Transmission Electron Microscopy image of core/shell nanoparticles and (**B**) corresponding histogram. (**C**) Transmission Electron Microscopy image of decorated nanoparticles and (**D**) corresponding histogram. (**E**) Expansion of image **C** (white square) and (**F**) corresponding histogram.

Scanning electron microscopy – transmission mode (STEM) and EDS analysis

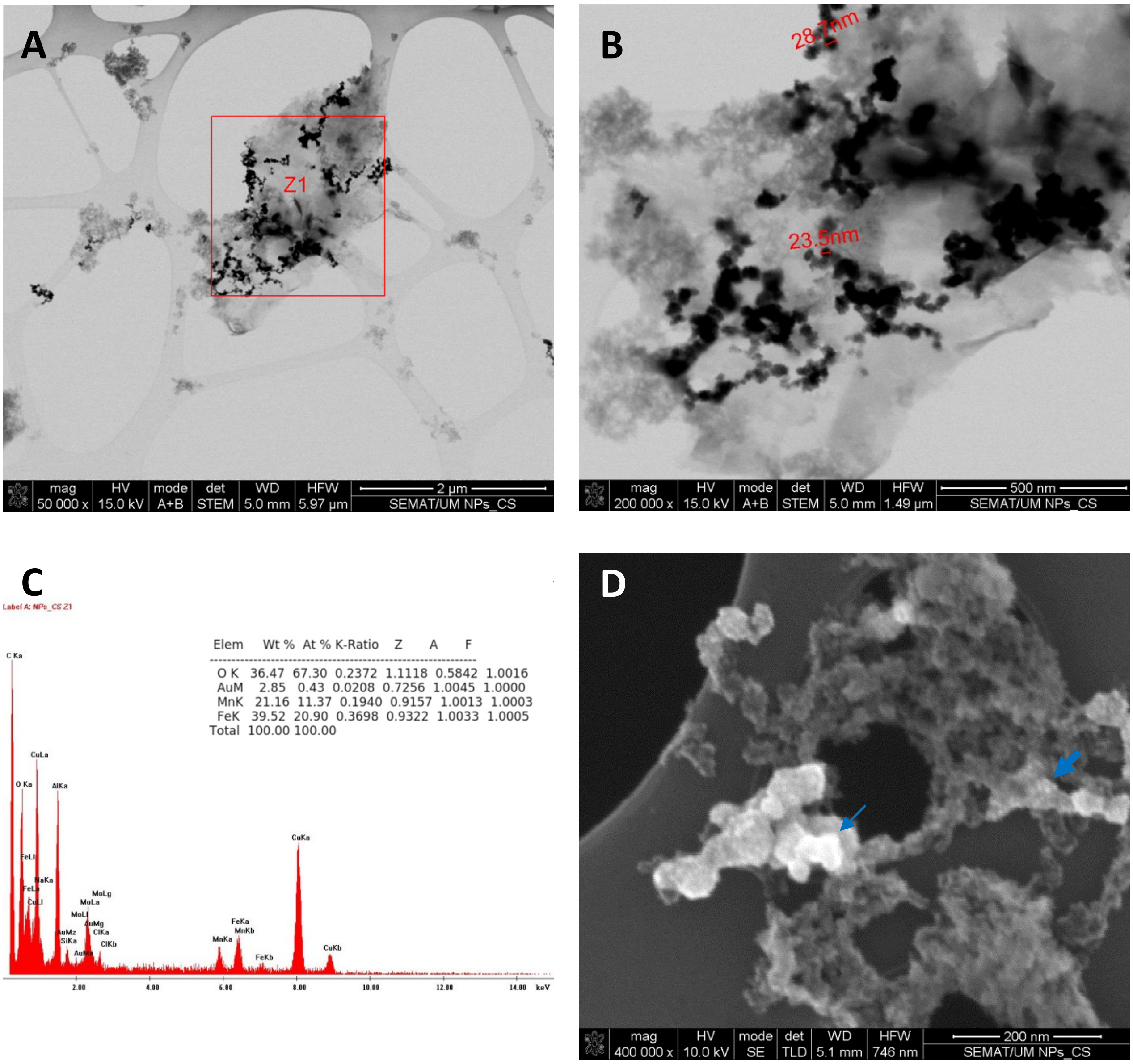

Figure S4. (**A**) and (**B**) Scanning electron microscopy images in transmission mode (STEM) of core/shell nanoparticles. (**C**) EDS spectrum of region Z1 (red square) from figure (**A**). (**D**) TLD image; particles covered by a gold homogeneous layer are marked with a thin blue arrow; particles with bright gold spots are marked with a thick blue arrow.
